# CuCrO_2_ Nanoparticles Incorporated into PTAA as a Hole Transport Layer for 85 °C and Light Stabilities in Perovskite Solar Cells

**DOI:** 10.3390/nano10091669

**Published:** 2020-08-26

**Authors:** Bumjin Gil, Jinhyun Kim, Alan Jiwan Yun, Kimin Park, Jaemin Cho, Minjun Park, Byungwoo Park

**Affiliations:** 1 Department of Materials Science and Engineering, Research Institute of Advanced Materials, Seoul National University, Seoul 08826, Korea; bestgil123@snu.ac.kr (B.G.); kim767@snu.ac.kr (J.K.); hangyeolee@snu.ac.kr (A.J.Y.); flamethrow@snu.ac.kr (K.P.); jjm7004@snu.ac.kr (J.C.); 2 Department of Chemical Engineering, Ulsan National Institute of Science and Technology, Ulsan 44919, Korea; sia835@unist.ac.kr

**Keywords:** perovskite solar cell, hole transport layer, CuCrO_2_ nanoparticles, thermal stability, light stability

## Abstract

High-mobility inorganic CuCrO_2_ nanoparticles are co-utilized with conventional poly(bis(4-phenyl)(2,5,6-trimethylphenyl)amine) (PTAA) as a hole transport layer (HTL) for perovskite solar cells to improve device performance and long-term stability. Even though CuCrO_2_ nanoparticles can be readily synthesized by hydrothermal reaction, it is difficult to form a uniform HTL with CuCrO_2_ alone due to the severe agglomeration of nanoparticles. Herein, both CuCrO_2_ nanoparticles and PTAA are sequentially deposited on perovskite by a simple spin-coating process, forming uniform HTL with excellent coverage. Due to the presence of high-mobility CuCrO_2_ nanoparticles, CuCrO_2_/PTAA HTL demonstrates better carrier extraction and transport. A reduction in trap density is also observed by trap-filled limited voltages and capacitance analyses. Incorporation of stable CuCrO_2_ also contributes to the improved device stability under heat and light. Encapsulated perovskite solar cells with CuCrO_2_/PTAA HTL retain their efficiency over 90% after ~900-h storage in 85 °C/85% relative humidity and under continuous 1-sun illumination at maximum-power point.

## 1. Introduction

In the field of next-generation photovoltaics, organic-inorganic hybrid halide perovskite solar cells have gathered tremendous attention since their emergence due to their rapidly growing power conversion efficiency (PCE), micrometer-scale carrier diffusion length, high absorption coefficient over solar spectrum regions, small exciton binding energy, etc. [1,2,3,4,5,6,7,8,9,10,11]. However, its relatively poor stability is still a main bottleneck toward commercialization, which becomes more serious at elevated temperatures or under constant illumination due to the rapid degradation of materials along with the accelerated formation and migration of defects [12,13,14,15,16,17,18]. One of the most vulnerable components is traditional organic small-molecule-based hole transport layers (HTL) such as 2,2′,7,7′-tetrakis(N,N-di-p-methoxyphenylamine)-9,9′-spirobifuorene (spiro-OMeTAD), which can easily decompose under the presence of heat [19,20]. Other candidates, such as poly(bis(4-phenyl)(2,5,6-trimethylphenyl)amine) (PTAA), are reported to be more durable in terms of stability [21,22,23,24,25], but diffusion of additives and ionic species can still occur to hamper the perovskite-HTL interface [26,27,28,29].

As an alternative to the unstable organic HTLs, inorganic HTLs such as CuSCN and various metal oxides have been shown to achieve long-term stability [30,31,32,33,34,35,36,37,38,39,40,41,42,43,44]. Among them, delafossite metal oxide CuCrO_2_ is considered as one of the most promising candidates as an HTL due to its high mobility of 0.1–1 cm^2^ V^−1^ s^−1^, favorable band alignment with perovskite, and facile synthesis method of nanoparticles by hydrothermal reaction of nitrate-based precursors [45,46,47,48,49,50,51]. Several research groups have adopted CuCrO_2_ HTL in a *p*-*i*-*n* structure to obtain ambient stability comparable to its organic counterparts [52,53,54,55]. However, few studies have utilized CuCrO_2_ in an *n*-*i*-*p* structure, mainly due to its difficulty in forming a uniform film over the perovskite layer [56]. Studies demonstrating long-term stabilities under continuous heat or light are also lacking; thus, a lot of effort is still required to successfully utilize CuCrO_2_ materials as a stable and efficient HTL.

One strategy to overcome the barrier of poor film formability of nanoparticle-type HTL is to co-utilize with other HTL that can form homogeneous precursor solutions, which can have multiple advantages over single-component solutions. The solution-based secondary HTL can successfully immerse between nanoparticles, which can greatly improve the film uniformity and thereby reduce surface/interface-related defects. The ability to utilize high-mobility nanoparticles can also improve the overall hole mobility of the HTL and the stability of the perovskite-HTL interface, especially when the solution-based HTL is known to be susceptible to the interfacial degradation. Several studies have demonstrated this hybrid-type design, such as NiO_x_/spiro-OMeTAD, NiO_x_/CuSCN, and CuGaO_2_/CuSCN, indicating the potential for further improvement of HTL by this co-utilization approach [57,58,59].

In this work, hydrothermally synthesized CuCrO_2_ nanoparticles are incorporated into the conventional PTAA to form CuCrO_2_/PTAA hybrid HTL that can effectively reduce the surface roughness. The utilization of high-mobility and stable CuCrO_2_ can boost the hole extraction while passivating deep-level traps, which are confirmed by optoelectronic analyses. Stabilities of ~900 h under 85 °C/85% relative humidity (RH) and continuous 1-sun illumination further confirm the successful durability of the bilayer HTL, suggesting a straightforward but effective method to improve the stabilities of perovskite solar cells.

## 2. Materials and Methods

### 2.1. Synthesis of CuCrO_2_ Nanoparticles

Cu(NO_3_)_2_·2.5H_2_O (Alfa Aesar, Heysham, UK) and Cr(NO_3_)_3_·9H_2_O (Alfa Aesar, Heysham, UK) were dissolved in deionized water (DW) with concentration of 0.21 M each. After 15 min of stirring, 1.8 M of NaOH (Daejung, Siheung, Korea) was added, and the solution was stirred for another 15 min. Then, the solution was transferred to a Teflon-lined stainless-steel autoclave and placed in an oven with a temperature of 220 °C for 60 h. After the reaction, a dark-green precipitate containing CuCrO_2_ nanoparticles was formed. The synthesized nanoparticles were centrifuged and sequentially washed with 1 N HCl (Daejung, Siheung, Korea) and isopropyl alcohol (IPA, Daejung, Siheung, Korea) four times, and stored in IPA for future use.

### 2.2. Device Fabrication

Glasses coated with indium-doped tin oxide (ITO) were cleaned in acetone (Daejung, Siheung, Korea), ethanol (Daejung, Siheung, Korea), and DW for 15 min each by sonication, followed by UV-ozone treatment for 15 min. For the electron transport layer, SnO_2_ aqueous colloidal dispersion (Alfa Aesar, Heysham, UK) was diluted by DW to 2.5 wt. %, spin-coated on ITO at 3000 rpm for 30 s, and annealed at 120 °C for 30 min. Perovskite precursor solution of 1.3 M Cs_0.05_(FA_0.85_MA_0.15_)_0.95_Pb(I_0.85_Br_0.15_)_3_ (FA and MA stand for formamidinium and methylammonium, respectively) was fabricated by dissolving PbI_2_ (TCI, Fukaya, Japan), PbBr_2_ (TCI, Fukaya, Japan), FAI (Greatcell Solar, Queanbeyan, Australia), MABr (Greatcell Solar, Queanbeyan, Australia), and CsI (TCI, Fukaya, Japan) with desired ratio in a 4:1 (*v*/*v*) mixture of N,N-dimethylformamide (DMF, Sigma-Aldrich, St. Louis, MO, USA) and dimethyl sulfoxide (DMSO, Sigma-Aldrich, St. Louis, MO, USA). In a N_2_-filled glovebox, the perovskite solution was deposited on a SnO_2_ layer by spin-coating at 1000 rpm for 10 s, followed by 5000 rpm for 20 s. A total of 300 μL of chlorobenzene (Sigma-Aldrich, St. Louis, MO, USA) was dripped onto the spinning substrate 3 s before the end of the spin-coating process. The samples were then annealed at 100 °C for 40 min. For the CuCrO_2_ hole transport layer, the stored CuCrO_2_ nanoparticle dispersion was further diluted by IPA to the desired concentrations (0.5–3 mg mL^−1^), subjected to sonication for 1 h, and spin-coated at 5000 rpm for 30 s, followed by annealing at 50 °C for 10 min to remove residual solvent. For a CuCrO_2_-only device, the spin-coating steps were repeated multiple times to obtain full coverage of HTL, whereas for a CuCrO_2_/PTAA device, single spin-coating of CuCrO_2_ was sufficient. For the PTAA hole transport layer, solution was fabricated by dissolving 20 mg of PTAA (47 kDa, MS Solutions, Seoul, Korea) in 1 mL of chlorobenzene, with the addition of 6 μL of 4-*tert*-butylpyridine (Sigma-Aldrich, St. Louis, MO, USA) and 4 μL of 520 mg mL^−1^ bis(trifluoromethane)sulfonimide lithium salt (Sigma-Aldrich, St. Louis, MO, USA) solution in acetonitrile (Sigma-Aldrich, St. Louis, MO, USA). The PTAA solution was then spin-coated on either perovskite film or pre-deposited CuCrO_2_ film at 3000 rpm for 30 s. Finally, an Au electrode was deposited by thermal evaporation. For encapsulated devices, the devices were sealed with cover glass using UV-curable epoxy resin (Nagase, Osaka, Japan).

### 2.3. Characterization

X-ray diffraction was conducted using a diffractometer (New D8 Advance, Bruker, Billerica, MA, USA). Surface roughness of the film was analyzed by an atomic force microscope (NX-10, Park Systems, Suwon, Korea). The cross-sectional image of an HTL film was obtained by a field-emission scanning electron microscope (Merlin-Compact, Zeiss, Oberkochen, Germany). The optical bandgap was analyzed by UV-visible absorption spectroscopy using a spectrophotometer (V-770, JASCO, Easton, MD, USA). A time-of-flight secondary ion mass spectrometer (TOF-SIMS-5, IONTOF, Münster, Germany) was utilized to obtain the depth profile of the device. Photoluminescence (LabRam HV Evolution, Horiba, Kyoto, Japan) and time-resolved photoluminescence (FluoTime 300, Picoquant, Berlin, Germany) of the films were analyzed using lasers with excitation wavelengths of 523 nm and 398 nm, respectively. Space-charge limited current (SCLC) and admittance analyses were conducted using a potentiostat (Zive SP-1, WonATech, Seoul, Korea), where dark current was measured under varying direct current (DC) bias for SCLC measurement and impedance was measured at a frequency of 10^−2^–10^4^ Hz using 10 mV AC voltage perturbation for admittance analysis. J-V curves of the solar cells were obtained using a solar simulator (K3000, McScience, Suwon, Korea) with 1-sun (AM 1.5G) illumination on the glass/ITO side, with a voltage sweep between 1.2 and −0.1 V, a scan rate of 100 mV s^−1^, and active areas for solar cells were 0.09 cm^2^. For the thermal stability test, encapsulated devices were stored within a dark test chamber (TH-PE-025, JeioTech, Daejeon, Korea) with controlled temperature and humidity (85 °C/85% RH), and J-V scans of devices were periodically conducted. For the light stability test, encapsulated devices were tested with maximum-power-point tracking equipment (K3600, McScience, Suwon, Korea) under continuous 1-sun illumination, where maximum-power voltage is constantly applied to the cells during the test.

## 3. Results and Discussion

**Figure 1:**
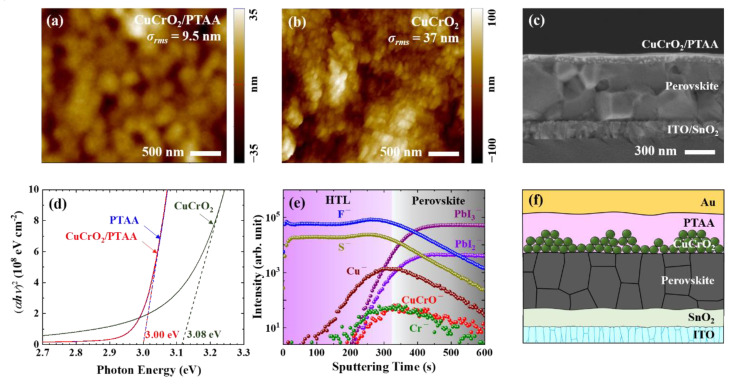
Morphological and structural analyses of CuCrO_2_/PTAA and CuCrO_2_ hole transport layer (HTL): (**a,b**) Topography and root-mean-square (RMS) surface roughness of each HTL deposited on indium-doped tin oxide (ITO)/SnO_2_/perovskite, obtained by atomic force microscope (AFM); (**c**) Cross-sectional scanning electron microscopy (SEM) image of ITO/SnO_2_/perovskite/HTL film; (**d**) UV-visible absorption spectra and the optical bandgap energy of each HTL deposited on a glass substrate; (**e**) Time-of-flight secondary ion mass spectrometry (TOF-SIMS) depth profile of the ITO/SnO_2_/perovskite/CuCrO_2_/PTAA film; (**f**) Schematic illustration of the device architecture with CuCrO_2_/PTAA HTL. Light is incident on an ITO side during the solar cell operation.

One of the prerequisites for HTL in an *n*-*i*-*p* type perovskite solar cell is continuous film formability that can yield a thin and compact layer above a perovskite substrate. The CuCrO_2_/PTAA HTL deposited on the conventional triple-cation perovskite (Cs_0.05_(FA_0.85_MA_0.15_)_0.95_Pb(I_0.85_Br_0.15_)_3_) displayed a smooth surface topography with reasonably small surface roughness (Figure 1a). However, due to the agglomeration of CuCrO_2_ nanoparticles when deposited on perovskite substrate, simple one-step spin-coating of CuCrO_2_ nanoparticles alone often yielded incomplete coverage; hence, multiple spin-coatings were required for CuCrO_2_ to fully cover the underlying perovskite layer (Figure S1a,b). Moreover, even though the full coverage was obtained with only CuCrO_2_, the resulting HTL displayed a much larger surface roughness compared to the CuCrO_2_/PTAA, resulting in a low PCE (Figure 1b and Figure S1c). The difficulty in creating a uniform film with nanoparticles alone suggests that incorporating a small number of nanoparticles within other solution-processable HTL is more suitable to utilize high-mobility nanoparticles, as suggested in this work. As shown in the cross-sectional image in Figure 1c, the final CuCrO_2_/PTAA bilayer showed compact and dense morphology with ~100 nm thickness, suggesting that the PTAA solution was effectively wetted and immersed among the CuCrO_2_ nanoparticles, and thereby formed a uniform film without any visible structural imperfections. Further analyses by x-ray diffraction (XRD) revealed that hydrothermally synthesized CuCrO_2_ nanoparticles consisted of a mixture of desirable rhombohedral and hexagonal delafossite phases without any detectable impurities (Figure S2a) [52], and the perovskite layer was not damaged or decomposed into impurities like PbI_2_ after the deposition of CuCrO_2_/PTAA HTL (Figure S2b).

The optical bandgap was determined to be 3.00 eV and 3.08 eV for PTAA-only and CuCrO_2_-only HTL, respectively, as presented in Figure 1d [45,46,48,49,50,60,61]. The absorption spectrum and bandgap of the optimized CuCrO_2_/PTAA layer were almost identical to those of PTAA, since a small number of CuCrO_2_ nanoparticles was enough to form an effective and stable HTL layer. The presence and distribution of CuCrO_2_ in the bilayer HTL was further confirmed by a time-of-flight secondary ion mass spectroscopy (TOF-SIMS), as shown in Figure 1e. PbI_2_^−^ and PbI_3_^−^ originated from the perovskite, and F^−^ and S^−^ originated from the additives of PTAA. Since species containing Cu and Cr were distributed near the perovskite-HTL interface, the CuCrO_2_ nanoparticles were mainly located at the bottom part of the HTL, where they were percolated by the solution-processed PTAA (Figure 1f).

**Figure 2:**
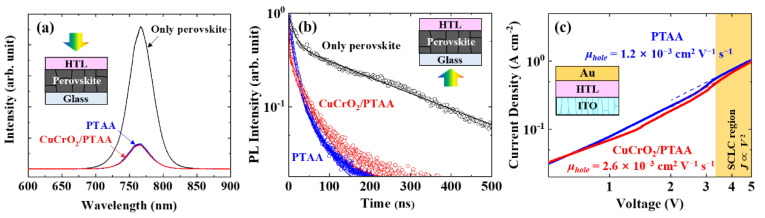
Electronic properties of CuCrO_2_/PTAA HTL: (**a**) Steady-state photoluminescence (PL) and (**b**) time-resolved PL spectra (398-nm excitation with fitting lines) of bare perovskite and perovskite/HTL films deposited on glass; (**c**) Dark J-V characteristics of ITO/HTL/Au layers with different HTLs, where space-charge limited current (SCLC) region is indicated.

The electronic properties of CuCrO_2_/PTAA hybrid HTL were investigated to evaluate its ability to extract and transport holes. Photoluminescence (PL) spectra in Figure 2a show that CuCrO_2_/PTAA HTL exhibited slightly larger PL quenching compared to the bare PTAA, implying the increased hole-extracting ability due to the incorporation of high-mobility CuCrO_2_ nanoparticles. Time-resolved PL spectra, as seen in Figure 2b, exhibited faster early-stage decay with CuCrO_2_/PTAA compared to PTAA, 14 vs. 28 ns, respectively [62,63,64]. To characterize the hole-extracting mobility more quantitatively, dark current-voltage (J-V) characteristics under DC bias were examined for the SCLC region with the ITO/HTL/Au structure (Figure 2c) [65,66]. The hole mobilities were 2.6 × 10^−3^ and 1.2 × 10^−3^ cm^2^ V^−1^ s^−1^ for CuCrO_2_/PTAA and bare PTAA, respectively, further supporting the role of high-mobility CuCrO_2_ nanoparticles which enable faster hole extraction from the perovskite along the HTL.

**Figure 3:**
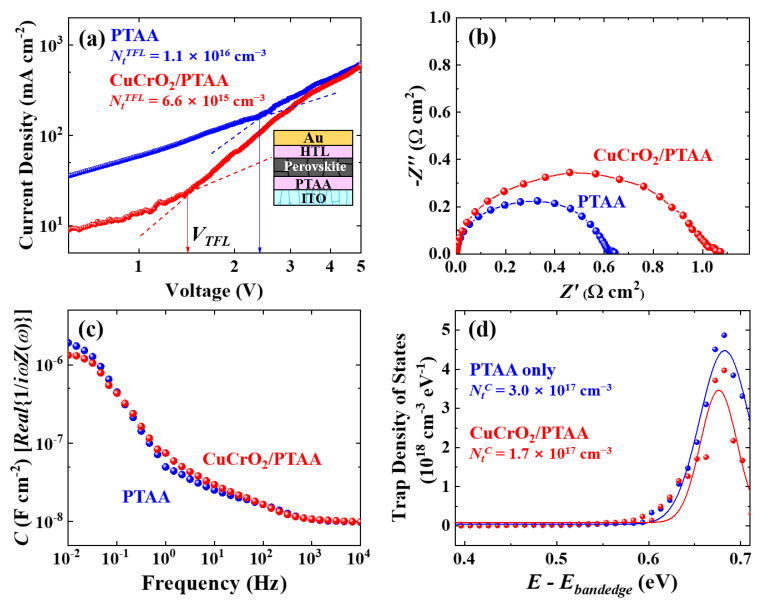
Trap density analyses of devices with different HTLs: (**a**) Dark J-V characteristics of hole-only devices with different upper HTLs, and calculated trap densities (N_t_^TFL^) from trap-filled limited voltages (V_TFL_); (**b**) Nyquist plot, (**c**) capacitance-frequency plot, and (**d**) trap density of states obtained from the capacitances (with the integrated trap density N_t_^C^), in the device structure of ITO/SnO_2_/perovskite/HTL/Au.

Next, the effect of CuCrO_2_ incorporation into PTAA on the defect characteristics of the devices is discussed. Figure 3a shows dark J-V characteristics of hole-only devices with the structure of ITO/PTAA/perovskite/HTL/Au, with the upper HTL being either PTAA or CuCrO_2_/PTAA. The trap-filled limited voltages (V_TFL_) are related to the trap densities of the devices (N_t_^TFL^), exhibiting 6.6 × 10^15^ and 1.1 × 10^16^ cm^−3^ for the CuCrO_2_/PTAA and bare PTAA, respectively [67]. Defect densities were also analyzed by capacitance analyses on the conventional ITO/SnO_2_/perovskite/HTL/Au solar-cell structures. Nyquist plots in Figure 3b show larger semicircles for the device with CuCrO_2_/PTAA compared to the PTAA, implying the increased recombination resistance which may be related to the decreased trap sites along the perovskite-HTL region [68,69,70,71]. It can also be seen in Figure 3c that two devices show different capacitive responses at low frequencies, indicating differences in the midgap trap states [68,72,73]. The trap density of states derived from the derivative of the capacitance (Figure 3d) exhibited a lower density of states in CuCrO_2_/PTAA HTL, resulting in an almost halved integrated trap density (N_t_^C^) compared to the PTAA HTL [74,75,76]. These combined results of trap reduction suggest that CuCrO_2_ nanoparticles near the perovskite-HTL interface surely passivate defects and related trap states, which can also contribute to the improvement of charge transport, as previously mentioned.

**Figure 4:**
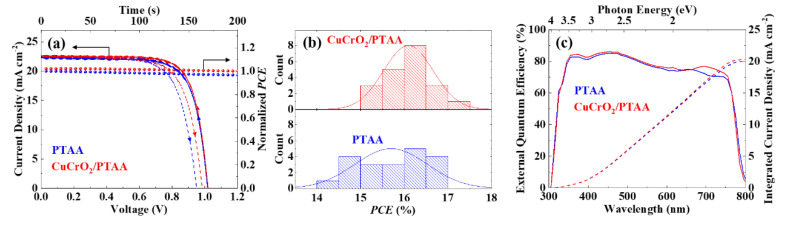
Photovoltaic performances of perovskite solar cells with CuCrO_2_/PTAA or PTAA as HTL: (**a**) J-V curves of champion cells and their steady-state efficiencies under maximum power voltage; (**b**) PCE distributions for 20 cells at each condition; (**c**) External quantum efficiency (EQE) of solar cells.

Solar cells with the device structure of ITO/SnO_2_/perovskite/HTL/Au were fabricated with either CuCrO_2_/PTAA or PTAA. With the optimum concentration of CuCrO_2_ nanoparticles (Figure S3a), the champion cell yielded V_OC_ = 1.02 V, J_SC_ = 22.8 mA cm^−2^ and FF = 0.75 (PCE of 17.4%), whereas the bare PTAA yielded V_OC_ = 1.02 V, J_SC_ = 22.4 mA cm^−2^ and FF = 0.74 (PCE of 16.9%), as shown in Figure 4a (with the device parameters for multiple cells presented in Table 1 and Figure S3). While the external quantum efficiencies (EQEs) of the devices were quite similar (Figure 4c), the response at a longer wavelength (~700 nm or ~1.7 eV) exhibited better efficiency with the CuCrO_2_-nanoparticle device, indicating an improved hole carrier collection, consistent with Figure 2 and Figure 3. This improved hole collectivity might contribute to the increase of average J_SC_, whereas the slight increases in trap-dependent parameters such as V_OC_ and FF further confirm the defect passivation effect by CuCrO_2_ nanoparticles at the perovskite-HTL interface [77,78].

**Figure 5:**
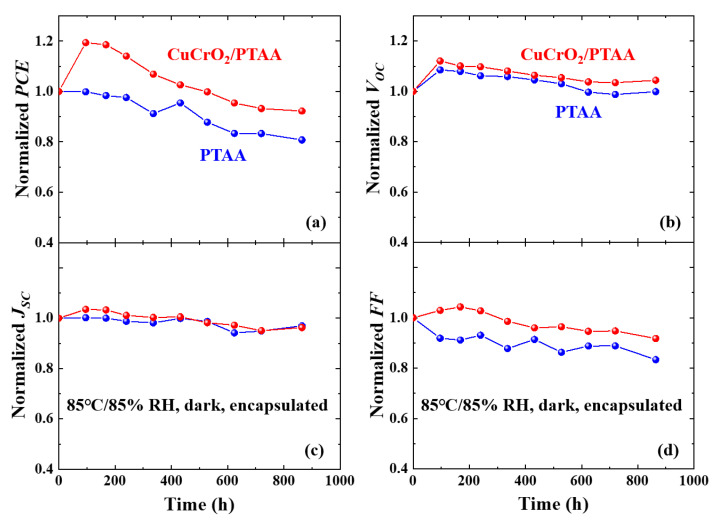
Thermal stabilities of solar cells with CuCrO_2_/PTAA or PTAA HTL: Normalized values of (**a**) efficiency, (**b**) V_OC_, (**c**) J_SC_, and (**d**) FF of the encapsulated solar cells stored under 85 °C/85% relative humidity (RH) dark condition.

The effect of CuCrO_2_ nanoparticles on both thermal and light stabilities of the solar cells were also investigated. For thermal stability, encapsulated devices were stored under standard damp heat conditions (85 °C/85% relative humidity (RH)) [25,79,80], where encapsulation was applied to block other external degradation factors than heat. High humidity was used to detect devices with damaged encapsulation which would undergo rapid moisture-induced degradation with a leak. As presented in Figure 5, improved thermal stability was observed for the device with CuCrO_2_/PTAA HTL, where the device maintained over 90% of its initial PCE after 860 h. The degradation of organic cations in the perovskite or small organic molecules within HTL can critically damage both bulk and the interface, especially at an elevated temperature [9,10,20,81,82]. It can be inferred that the presence of more heat-resistant CuCrO_2_ nanoparticles in the vicinity of perovskite and HTL creates a more heat-resistant interface with reduced interfacial reactions to maintain excellent thermal stability.

**Figure 6:**
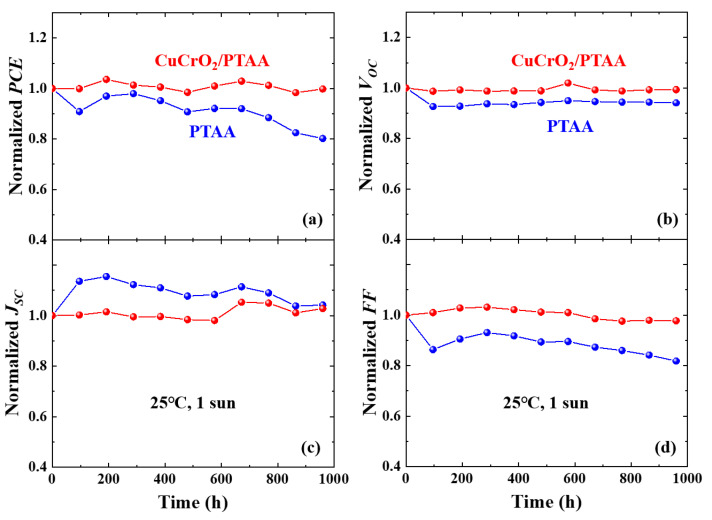
Light stabilities of solar cells with CuCrO_2_/PTAA or PTAA HTL: Normalized values of (**a**) efficiency, (**b**) V_OC_, (**c**) J_SC_, and (**d**) FF of the encapsulated solar cells obtained by maximum-power-point tracking (MPPT) under continuous illumination of AM 1.5G at 25 °C.

Light stabilities were tested by maximum-power-point tracking (MPPT) under continuous 1-sun (AM 1.5G) illumination. Figure 6 shows that a solar cell adopting CuCrO_2_/PTAA HTL retains almost the entirety of its initial PCE after 960 h of operation, demonstrating superior light stability over the bare-PTAA device. Migration of halide defects as well as Li^+^ from the additive in PTAA can accumulate at the perovskite-HTL interface and trigger interfacial degradation under operation conditions [15,28,29,83]. The improved light stability confirms that CuCrO_2_ nanoparticles can directly prevent potential accumulation of traps at the interface, resulting in superior solar cells.

## 4. Conclusions

Hybrid HTL, consisting of high-mobility CuCrO_2_ nanoparticles embedded between perovskite and PTAA, was facilely adopted in the perovskite solar cells to guarantee excellent thermal and light stabilities. By a simple solution process, CuCrO_2_/PTAA HTL was fabricated, yielding a uniform and smooth morphology. With CuCrO_2_ nanoparticles providing high-mobility charge transport paths, CuCrO_2_/PTAA HTL demonstrated more efficient hole-extraction abilities than the bare PTAA, and trap density was reduced by nearly half with CuCrO_2_ nanoparticles. Therefore, solar cells with bilayer CuCrO_2_/PTAA yielded higher PCE than the conventional PTAA-based ones, and also maintained over 90% of the initial efficiencies after storage under 85 °C/85% RH or operating under 1-sun MPPT for ~900 h. Our novel design of organic-inorganic hybrid HTL can aid in developing perovskite-based devices with improved hole extractability and reduced defects/traps, which ultimately leads to the superior stabilities under thermally induced and light-induced conditions.

## Figures and Tables

**Table 1:** Photovoltaic parameters of the solar cells (reverse scan for 20 cells). The data in parentheses are from the cells with the best power conversion efficiency (PCE).

**HTL**	**V_OC_ (V)**	**J_SC_** **(mA cm^−2^)**	**FF**	**PCE (%)**	**HI** **(1–η_FOR_/η_REV_) ^1^**
PTAA	1.02 ± 0.03 (1.02)	21.3 ± 1.1 (22.4)	0.72 ± 0.02 (0.74)	15.7 ± 0.8 (16.9)	0.09 ± 0.04
CuCrO_2_/PTAA	1.03 ± 0.15 (1.02)	21.6 ± 3.3 (22.8)	0.73 ± 0.11 (0.75)	16.1 ± 2.4 (17.4)	0.11 ± 0.04

^1^ HI, η_FOR_ and η_REV_ refer to the hysteresis index, forward-scan PCE and reverse-scan PCE, respectively.

## Supplementary Materials

The following are available online at https://www.mdpi.com/2079-4991/10/9/1669/s1. Figure S1: Solar cells adopting only CuCrO_2_ as an HTL; Figure S2: X-ray diffraction of CuCrO_2_ nanoparticles and perovskite/CuCrO_2_/PTAA HTL; Figure S3: Performances of solar cells adopting either CuCrO_2_/PTAA or PTAA as an HTL.
