# Supplementary material for: CuCrO2 Nanoparticles Incorporated into PTAA as a Hole Transport Layer for 85 °C and Light Stabilities in Perovskite Solar Cells
Source: Nanomaterials (Basel). 2020 Aug 26;10(9):1669. doi: 10.3390/nano10091669 (PMC7558584; doi:10.3390/nano10091669)
Supplement: Supplementary file 1 [file nanomaterials-10-01669-s001.pdf]

nanomaterials

Supplementary Materials

$CuCrO_2$ Nanoparticles Incorporated into PTAA as a Hole Transport Layer for 85°C and Light Stabilities in Perovskite Solar Cells

Bumjin Gil [1,†], Jinhyun Kim [1,†], Alan Jiwan Yun [1], Kimin Park [1], Jaemin Cho [1], Minjun Park [2] and Byungwoo Park [1,*]

[1] Department of Materials Science and Engineering, Research Institute of Advanced Materials, Seoul National University, Seoul 08826, Republic of Korea; bestgil123@snu.ac.kr (B.G.); kim767@snu.ac.kr (J.K.); hangyeolee@snu.ac.kr (A.J.Y.); flamethrow@snu.ac.kr (K.P.); jjm7004@snu.ac.kr (J.C.)
[2] Department of Chemical Engineering, Ulsan National Institute of Science and Technology, Ulsan 44919, Republic of Korea; sia835@unist.ac.kr (M.P.)
* Correspondence: byungwoo@snu.ac.kr (B.P.)
† These authors contributed equally to this work.

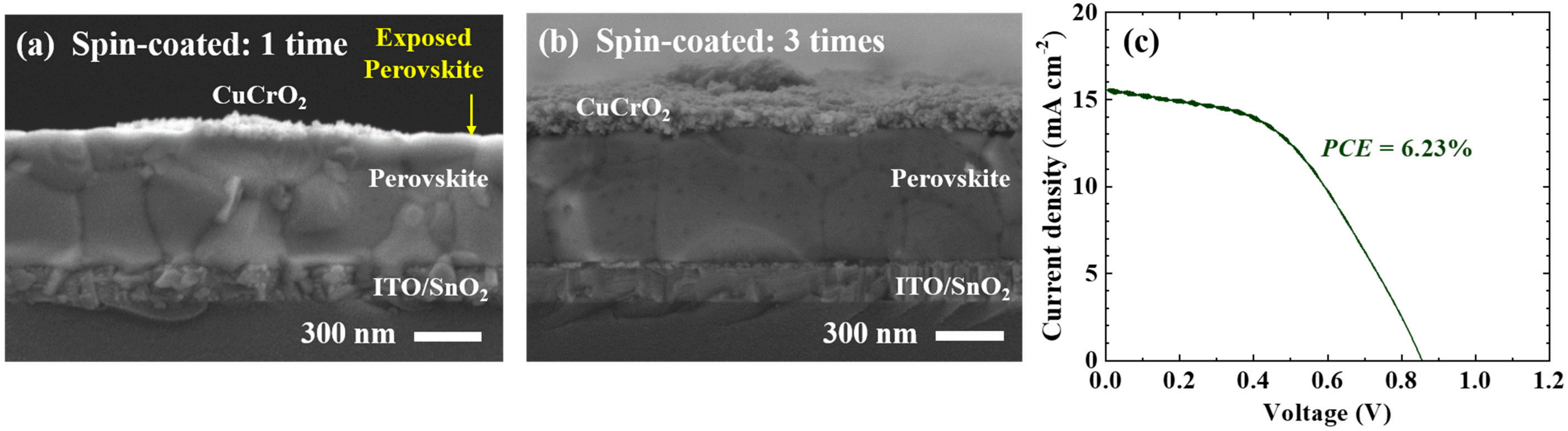

Figure S1. Solar cells adopting only $CuCrO_2$ as an HTL: (**a**,**b**) Cross-sectional scanning electron microscope (SEM) images of ITO/SnO_2/perovskite/$CuCrO_2$ films where $CuCrO_2$ nanoparticle solution is spin-coated on the perovskite 1 time and 3 times, respectively. The yellow arrow indicates exposed perovskite; (**c**) A J-V curve of the device using only $CuCrO_2$ as an HTL. The optimized number of spin coating is 3 times, where the device parameters are: V_{OC} = 0.855 V, J_{SC} = 15.58 mA cm^{-2}, FF = 0.471, and PCE = 6.23%.

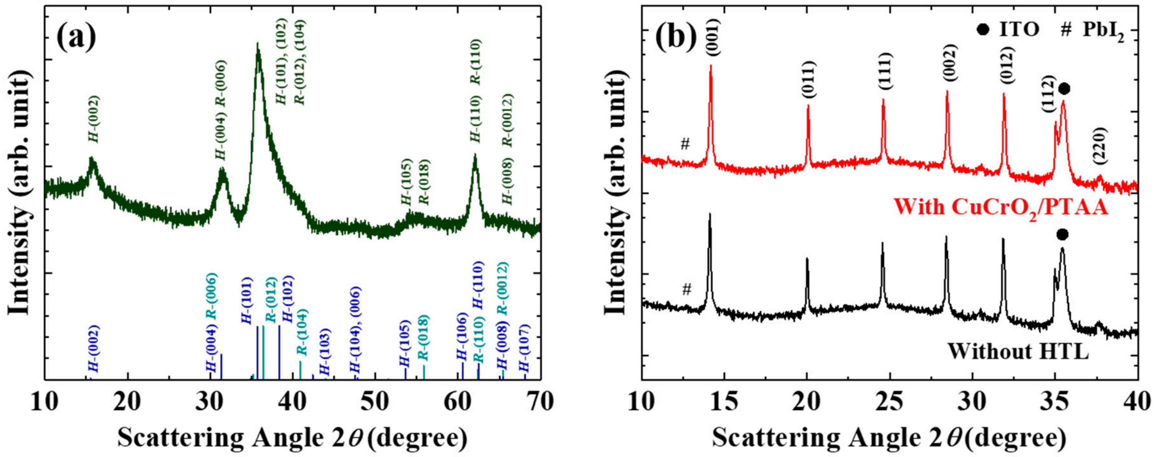

Figure S2. X-ray diffraction of $CuCrO_2$ nanoparticles and perovskite/$CuCrO_2$/PTAA HTL: (**a**) *R* and *H* stand for the rhombohedral (ICDD 01-074-0983) and hexagonal (ICDD 04-070-0746) $CuCrO_2$, respectively; (**b**) $Cs_{0.05}(FA_{0.85}MA_{0.15})_{0.95}Pb(I_{0.85}Br_{0.15})_3$ perovskite films with and without $CuCrO_2$/PTAA HTL.

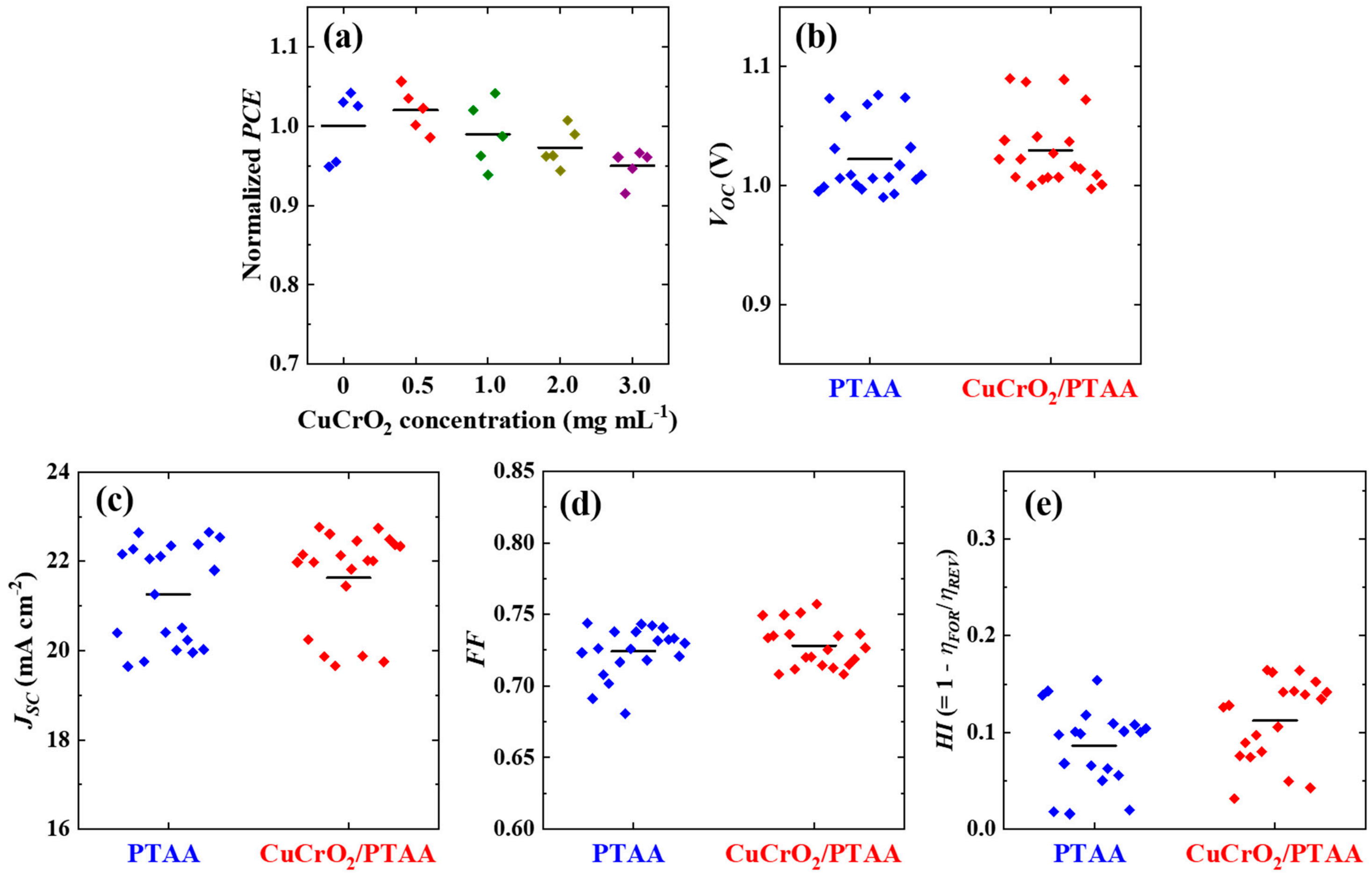

Figure S3. Performances of solar cells adopting either $CuCrO_2$/PTAA or PTAA as an HTL: (**a**) Efficiencies of solar cells using $CuCrO_2$/PTAA as an HTL with varying $CuCrO_2$ concentrations in the precursor dispersion; (**b**) V_{OC}, (**c**) J_{SC}, (**d**) FF, and (**e**) hysteresis index (HI = 1 − η_{FOR}/η_{REV}) of solar cells with the optimized $CuCrO_2$/PTAA vs. PTAA, measured for 20 devices at each condition (black lines representing average values).
